# Rapid Re-Union of Parts

**Published:** 1844-02-24

**Authors:** 


RAPID RE-UNION OF PARTS.

Dr. Centofanti, of Pisa, vouches for the truth of the following occurrence, in a Florentine medical journal:—A girl, fifteen years old, had two of her fingers suddenly chopped off, through the first phalanx, and without waiting to "pick up the pieces," she ran to seek surgical assistance. The surgeon caused immediate search to be made for the lost members, and was not a little surprised when they were presented to him doubly divided—that is, they had each been chopped into two pieces, the hand having, doubtless, been closed at the time of the accident. Notwithstanding this, the parts were replaced in their natural situation and closely invested with a bandage. In a few days adhesive inflammation was found to have clearly progressed; healing, in fact, proceeded rapidly, and in the course of the next year it was stated that the fingers had *regained their original mobility!*—*Lon. Lancet, from Omodei's Annali. Univ. di Med.*

